# New Record of *Metarhizium brunneum* Infecting Banana Weevil in Peru: Implications for Biological Control

**DOI:** 10.3390/jof12050363

**Published:** 2026-05-15

**Authors:** Edwin Mondragon-Herrera, Laydy Mitsu Mena-Chacon, Santos T. Leiva-Espinoza, Angel F. Huaman-Pilco

**Affiliations:** 1 Facultad de Ingeniería y Ciencias Agrarias, Universidad Nacional Toribio Rodríguez de Mendoza de Amazonas (UNTRM), Chachapoyas 01001, Peru; 7085037121@untrm.edu.pe; 2 Grupo de Investigación en Patología Intracelular de Plantas, Instituto de Investigación para el Desarrollo Sustentable de Ceja de Selva, Universidad Nacional Toribio Rodríguez de Mendoza de Amazonas, Chachapoyas 01001, Peru; laydy.mena@untrm.edu.pe; 3 Grupo de Investigación en Biopesticidas y Bioalternativas para la Protección Vegetal, Instituto de Investigación para el Desarrollo Sustentable de Ceja de Selva, Universidad Nacional Toribio Rodríguez de Mendoza de Amazonas, Chachapoyas 01001, Peru

**Keywords:** biological control agents, entomopathogenic fungi, conidial production, integrated pest management

## Abstract

The use of entomopathogenic fungi as biological control agents has gained increasing relevance as a sustainable alternative to chemical insecticides in tropical agroecosystems. In this study, a naturally occurring isolate of *Metarhizium brunneum* infecting adults of *Metamasius hemipterus* was recovered from banana plantations in the Amazonas region, Peru, and evaluated for its potential as a biological control agent. Multilocus phylogenetic analysis based on *tef1α*, *β-tubulin*, *rpb1*, and *rpb2* sequences confirmed its taxonomic identity within the *M. brunneum* clade. Physiological characterization revealed variability in growth and thermal response among isolates, while conidial production differed significantly depending on the substrate. Notably, isolate PM9 exhibited the highest conidial yield on rice substrate. Pathogenicity assays demonstrated high virulence against adult weevils, with an LC_50_ of 2.91 × 10^5^ conidia·mL^−1^ and mortality exceeding 90% at the highest concentration tested. These findings indicate that isolate PM9 combines desirable physiological and pathogenic traits for biological control. The natural occurrence of this entomopathogen in banana systems suggests ecological adaptation to local conditions and supports its potential incorporation into integrated pest management strategies, although further field-based evaluation is required.

## 1. Introduction

*Metarhizium brunneum* (Hypocreales: Clavicipitaceae) is a cosmopolitan entomopathogenic fungus widely recognized for its role as a biological control agent (BCA) against a broad range of insect pests. Species within the genus *Metarhizium* are among the most extensively studied fungal BCAs due to their infective capacity, ecological adaptability, and compatibility with integrated pest management (IPM) programs [1,2,3,4]. Members of this genus typically produce green conidia on the cadavers of infected arthropods, a characteristic that has led to their common designation as “green muscardine fungi” [5]. Most species have been isolated from soil environments or in association with plant roots, although they are occasionally recovered from naturally infected arthropod hosts [4,6].

Several studies have demonstrated the pathogenic potential of *M. brunneum* against economically important agricultural pests. Documented targets include the coffee leaf miner (*Leucoptera coffeella*) [7], the western corn rootworm (*Diabrotica virgifera virgifera*) [8], wireworms (*Agriotes* spp.) [9], the sugar beet weevil (*Asproparthenis punctiventris* [10], and the two-spotted spider mite (*Tetranychus urticae*) [11]. Additionally, secondary metabolites produced by this fungus have shown antifungal activity against plant pathogens such as *Verticillium dahliae* and *Phytophthora megasperma* [12]. However, despite promising laboratory results, field efficacy may vary depending on environmental conditions, formulation strategies, host susceptibility, and application methods [3,13].

From an ecological perspective, the environmental safety of *M. brunneum* has also been investigated. High-throughput sequencing studies have shown that its application does not significantly disrupt native soil microbial communities, suggesting ecological compatibility with existing microbiomes [8,14,15]. Moreover, interactions with other biological control agents appear generally compatible, although sublethal effects on certain beneficial insects have been reported, highlighting the importance of evaluating multitrophic interactions when integrating microbial agents into IPM programs [16].

In tropical agroecosystems, banana (*Musa* spp.) production is significantly affected by curculionid pests. Among them, *Metamasius hemipterus* (L.) (Coleoptera: Curculionidae), commonly known as the sugarcane weevil, has emerged as an important pest in banana plantations. Larvae and adults feed within pseudostems and corm tissues, causing structural damage that leads to plant weakening, lodging, and yield reduction [17,18,19]. The damage caused by this species can be comparable to that produced by the banana weevil *Cosmopolites sordidus*, and both pests may coexist within the same production systems, intensifying economic losses [20].

Entomopathogenic fungi have been investigated as sustainable alternatives for managing *M. hemipterus*. For example, *Metarhizium anisopliae* has demonstrated high virulence against this pest under laboratory conditions, particularly at elevated conidial concentrations [21], supporting the potential of *Metarhizium* spp. as biological control agents against curculionid pests. Nevertheless, information regarding the natural occurrence and pathogenicity of *M. brunneum* infecting *M. hemipterus* in banana agroecosystems remains scarce, especially in South American production regions.

Therefore, the objective of this study was to isolate and molecularly identify *Metarhizium brunneum* naturally infecting *Metamasius hemipterus* in banana crops in Amazonas, Peru, representing the first report of this host–pathogen association in the country. Subsequently, the entomopathogen was cultured, mass-produced on rice substrate, and evaluated in dose–response bioassays against adult *M. hemipterus* under controlled laboratory conditions.

## 2. Materials and Methods

### 2.1. Isolation and Establishment of Fungal Isolates

**Figure 1:**
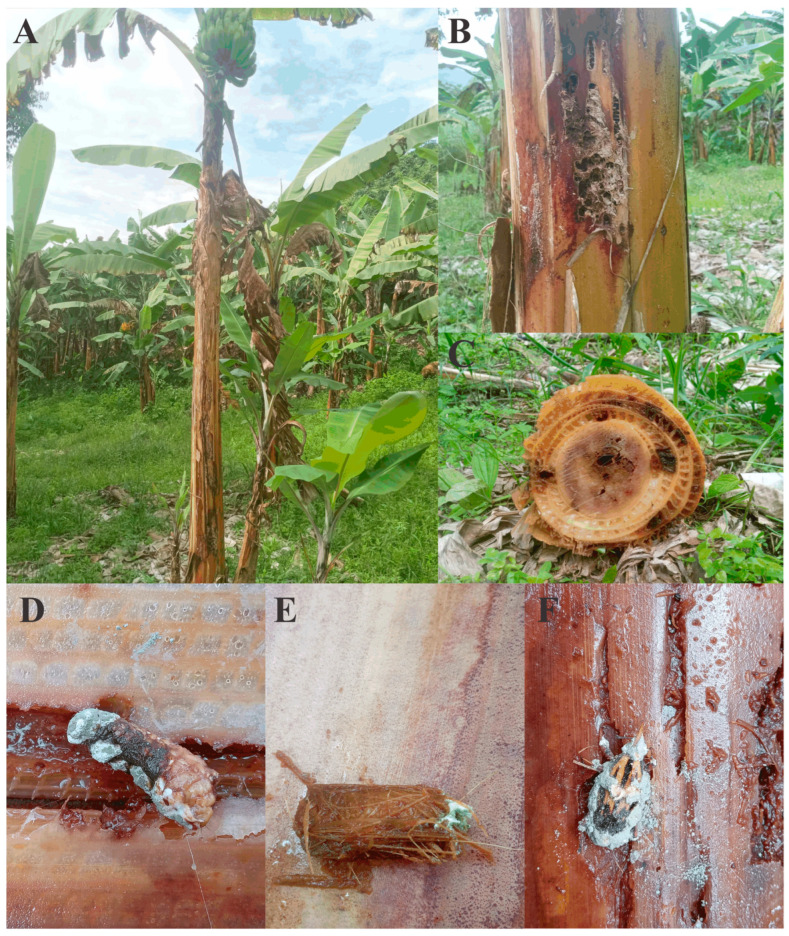
Field symptoms of *Metamasius hemipterus* infestation and natural infection by *Metarhizium brunneum* in banana plantations. (**A**) Banana plant showing severe pseudostem damage associated with weevil infestation. (**B**) External lesions and galleries in the pseudostem caused by *M. hemipterus* feeding activity. (**C**) Cross-section of banana pseudostem showing internal tunneling typical of weevil attack. (**D**–**F**) Naturally infected individuals of *M. hemipterus* displaying mycosis with characteristic white fungal growth consistent with *Metarhizium brunneum*.

Larvae, pupae, and adults of *Metamasius hemipterus* exhibiting external signs of mycosis consistent with *Metarhizium brunneum* infection (Figure 1) were collected from banana plantations located in the Amazonas region, Peru (Table 1). Infected specimens were surface-sterilized in 0.5% sodium hypochlorite (NaClO) for 3 min and rinsed thoroughly with sterile distilled water. Sterilized individuals were placed separately in moist chambers and incubated at 25 °C for 7 days to promote sporulation. Emerging conidia were transferred onto potato dextrose agar (PDA) for fungal propagation. To ensure genetic uniformity, monosporic isolates were obtained from polysporic cultures using the single-spore isolation technique [22].

### 2.2. Molecular Identification and Phylogenetic Analysis

Genomic DNA was extracted from 8-day-old cultures grown on PDA using the Wizard^®^ Genomic DNA Purification Kit (Promega, Madison, WI, USA), following the manufacturer’s protocol.

Four gene regions were amplified by PCR following the manufacturer’s protocol: translation elongation factor 1-alpha (*tef1a*) using primers EF1A-983F/EF1A-2218R, *β-tubulin (β-TUB)* using Bt2a/Bt2b, and the largest and second largest subunits of RNA polymerase II (*rpb1* and *rpb2*) using RPB1Af/RPB1Cr and RPB2-5F/RPB2-7Cr, respectively [23,24]. PCR products were sequenced by Macrogen Inc. (Seoul, Republic of Korea).

Forward and reverse sequences were edited, assembled, and manually inspected to generate consensus sequences. Representative sequences of closely related *Metarhizium* species, including type and reference strains, were retrieved from GenBank and incorporated into the dataset (Table 2). Multiple sequence alignments were performed using the MUSCLE algorithm [25] implemented in MEGA version 11 [26]. The four loci were concatenated into a multilocus dataset using SeaView version 5.0 [27]. jModelTest v2 was used to identify the most appropriate nucleotide substitution model under the Akaike Information Criterion (AIC) [28].

Phylogenetic relationships were inferred under the Maximum Likelihood (ML) criterion using the CIPRES Science Gateway platform [29]. Branch support was assessed by bootstrap analysis with 1000 replicates. The resulting phylogenetic tree was midpoint-rooted and visualized and edited using Itol v5 [30]. The sequences generated in this study were deposited in GenBank under accession numbers provided in Table 2.

### 2.3. Morphological Characterization

For macromorphological characterization, a conidial suspension (1 × 10^7^ conidia·mL^−1^) was prepared for each isolate. An aliquot of 80 µL was evenly distributed onto PDA plates using sterile borosilicate glass beads and incubated at 25 °C in darkness for 3 days to promote uniform colony establishment. Subsequently, 5-mm-diameter mycelial plugs were transferred to the center of 90-mm Petri dishes containing fresh PDA.

Colony morphology was recorded after 14 days of incubation at 25 °C and 30 °C. The evaluated traits included colony color, margin characteristics, and surface texture, following the descriptive criteria proposed by Baró et al. [31].

Because all isolates were molecularly identified as *Metarhizium brunneum*, microscopic examination was conducted to confirm morphological consistency within the species. Actively sporulating 5-mm PDA plugs were transferred onto fresh PDA plates and covered with sterile coverslips to facilitate the development of conidiophores and conidia in situ. Plates were incubated in moist chambers at 25 °C for 4 days.

Microscopic structures were examined using a Leica^®^ light microscope (Leica Microsystems, Wetzlar, Germany). Digital micrographs were captured, and morphometric analysis was performed using Image Tool version 3.0. The length and width of 50 randomly selected conidia per isolate were measured to determine conidial dimensions.

### 2.4. Physiological Characterization

#### 2.4.1. Mycelial Growth

Colony diameter was measured along four perpendicular axes (two orthogonal diameters), and the mean value was calculated for each replicate to minimize measurement bias due to irregular colony margins. The inoculated plates were incubated at 25, 30, and 35 ± 1 °C to evaluate the thermal tolerance of the fungus [22]. The effect of temperature on radial growth was expressed as the thermal sensitivity (*TS*, %) relative to 25 °C, calculated after 14 d using the Formula (1).

(1) $$TS\;(\%)=\frac{{CR}_{T}-{CR}_{25}}{{CR}_{25}}\times 100,$$

where *CR_T_* represents colony radial growth at the evaluated temperature (30 or 35 °C), and *CR_25_* represents colony radial growth at 25 °C. Positive *TS* values indicate growth stimulation, whereas negative values indicate growth inhibition relative to 25 °C.

#### 2.4.2. Conidial Production on PDA

For conidial production, 5-mm-diameter plugs were excised from the actively growing margin of 5-day-old colonies using a sterile cork borer and transferred to fresh PDA plates. Cultures were incubated at 25 ± 1 °C in darkness for 15 days. Four replicates were established per isolate. Conidia were harvested by flooding each plate with sterile distilled water containing 0.05% Tween 20 and gently scraping the colony surface with a sterile scalpel. The resulting suspension was homogenized for 1 min using a magnetic stirrer and filtered through sterile medical gauze to remove mycelial fragments [32]. Conidial production (CP) was quantified using a Neubauer hemocytometer. The concentration of conidia (conidia·mL^−1^) was calculated using the Formula (2).

(2) $$C=(Cc)(4\times 10^{6})\left[\frac{Fd}{80}\right],$$

where *Cc* represents the mean number of conidia counted in five large squares of the hemocytometer, and *Fd* corresponds to the dilution factor. Four independent counts were performed per isolate.

### 2.5. Conidial Production on Rice Substrate

Conidial production was evaluated using rice as a solid substrate. Polypropylene bags containing 30 g of rice were moistened with 10 mL of sterile distilled water and sterilized at 120 °C for 20 min. After cooling to room temperature, each bag was inoculated with 5 mL of a conidial suspension adjusted to 1 × 10^7^ conidia·mL^−1^. Four independent replicates were established per isolate. Inoculated rice bags were incubated at 25 ± 1 °C in darkness for 15 days to allow fungal colonization and sporulation. After incubation, the entire 30 g of colonized rice was transferred into 150 mL of sterile distilled water containing 0.05% Tween 20. The suspension was agitated on an orbital shaker for 30 min to dislodge conidia from the substrate and subsequently filtered through sterile medical gauze to remove rice debris and mycelial fragments [22,32].

Conidial concentration was determined using a Neubauer hemocytometer under a light microscope. Conidial yield was expressed as the number of conidia per gram of rice (conidia·g^−1^), based on the total suspension volume recovered. Four independent counts were performed per isolate.

### 2.6. Dose–Response Bioassay in Metamasius hemipterus Adults

Dose–response bioassays were conducted to determine the median lethal concentration (LC_50_) of isolate PM9, selected based on its high conidial production on rice substrate, follow the procedure described by Mayo-Hernández et al. [22], with slight modifications. Conidial suspensions were prepared at four concentrations: 1 × 10^5^, 1 × 10^6^, 1 × 10^7^, and 1 × 10^8^ conidia·mL^−1^. Experimental units consisted of 500-mL plastic containers with modified lids to allow ventilation. Each container was provisioned with pieces of banana pseudostem surface-sterilized in 0.5% sodium hypochlorite (NaClO) for 1 min and rinsed with sterile distilled water. A moistened cotton plug was added to maintain humidity.

Ten field-collected adults of *M. hemipterus* were introduced into each experimental container after surface sterilization in 0.5% NaClO for 1 min and rinsing with sterile distilled water. Adults were individually immersed in the corresponding conidial suspension for 30 s (dip method) and then transferred to the containers. Each treatment consisted of four independent replicates (10 insects per replicate; total *n* = 40 insects per treatment), including a control treatment with sterile distilled water containing 0.05% Tween 20 [33]. Containers were maintained at 26 ± 1 °C under a 12 h photoperiod for 12 days. Mortality was recorded daily. Dead insects were removed, surface-sterilized (70% ethanol for 30 s followed by 0.5% NaClO for 1 min), rinsed with sterile distilled water, and placed in moist chambers at 26 °C to confirm mycosis. Mortality was attributed to fungal infection only when external sporulation was observed.

### 2.7. Statistical Analysis

Data were analyzed using one-way analysis of variance (ANOVA), followed by Tukey’s multiple comparison test at a 95% confidence level (*p* < 0.05), to assess differences among isolates or treatments when applicable. For dose–response bioassays, median lethal concentrations (LC_50_ and LC_90_) were estimated using a four-parameter log-logistic model (Formula (3)) implemented in the drc package in R software 4.4.1 (R Core Team, Vienna, Austria). The dose–response relationship was described by the following function:

(3) $$f\left(x\right)=c+\frac{d-c}{1+exp\left[b(\log\left(x\right)-\log\left(e\right))\right]}$$

where *x* is the conidial concentration, *c* and *d* represent the lower and upper asymptotes, respectively, *b* is the slope around the inflection point, and *e* corresponds to the median lethal concentration (LC_50_).

Model parameters were estimated by nonlinear regression. Model fit was evaluated based on residual analysis, root mean square error (RMSE), and significance of model parameters (*p* < 0.05). Goodness-of-fit was additionally examined by visual inspection of residual distribution and predicted versus observed values. All statistical analyses were performed in R version 4.4.1.

## 3. Results

### 3.1. Molecular Identification of the Isolates

**Figure 2:**
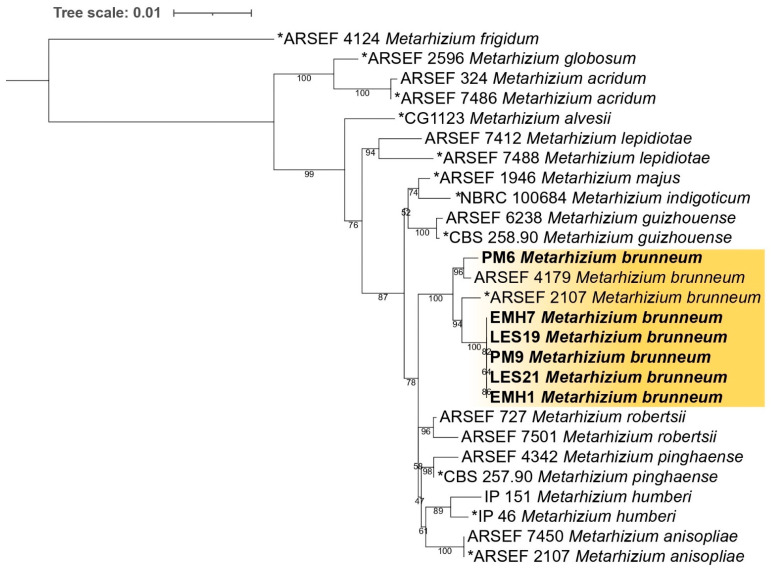
Multilocus phylogenetic tree of *Metarhizium* species. Maximum Likelihood phylogenetic tree inferred from concatenated TEF-1α, β-TUB, RPB1, and RPB2 sequences. Isolates obtained in this study are shown in bold and cluster within the *Metarhizium brunneum* clade together with reference strains ARSEF 4179 and the ex-type strain ARSEF 2107T. The tree was midpoint-rooted. “*” indicates ex-type/reference strains.

Multilocus phylogenetic analysis was performed under the TIM3 + I + G substitution model (transition model with unequal base frequencies, proportion of invariable sites, and gamma-distributed rate heterogeneity). The concatenated *tef1a*, *β-tubulin (β-tub)*, *rpb1,* and *rpb2* sequences resolved the relationships among the studied isolates and representative reference strains within the genus *Metarhizium* (Figure 2). The combined dataset separated the major species-level clades with strong bootstrap support. All isolates obtained from *Metamasius hemipterus* clustered within the *Metarhizium brunneum* clade, together with reference strains ARSEF 4179 and the ex-type strain ARSEF 2107T. This grouping was supported by high bootstrap values (≥96%), confirming their taxonomic placement as *M. brunneum*.

The *M. brunneum* lineage was well resolved and distinct from closely related species, including *M. robertsii*, *M. anisopliae*, and *M. pinghaense*, which formed separate and strongly supported clades. No internal phylogenetic structuring or evidence of cryptic divergence was detected among the isolates within the *M. brunneum* cluster.

### 3.2. Characterization of Isolates

#### 3.2.1. Morphological Characteristics

**Figure 3:**
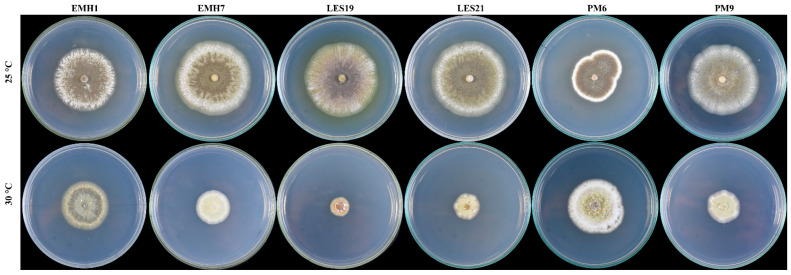
Colony morphology of *Metarhizium brunneum* isolates cultured on PDA at different temperatures. Representative colonies of isolates EMH1, EMH7, LES19, LES21, PM6, and PM9 grown on potato dextrose agar (PDA) at 25 °C (**upper row**) and 30 °C (**lower row**) after 14 days of incubation. Differences in radial growth, colony density, and aerial mycelium development are visible among isolates and between temperature conditions.

Colony morphology varied moderately among isolates when cultured on PDA at 25 °C and 30 °C (Figure 3). At 25 °C, all isolates developed well-expanded colonies with abundant aerial mycelium. Colonies were predominantly white to cream in color, with central zones exhibiting subtle beige to pale olive tones in some isolates. Margins were generally entire and well defined, although slight undulation was observed in isolates such as LES19 and PM6. Texture was mainly floccose to cottony, with evident radial growth patterns and moderate central density.

At 30 °C, overall radial growth was reduced in most isolates, and colonies exhibited a more compact morphology with decreased aerial mycelium (Figure 2). Surface coloration remained predominantly white to cream; however, central pigmentation became less evident compared to 25 °C. Margins were regular and sharply delimited, and colony texture shifted toward a denser and more compact appearance.

Isolate PM6 displayed comparatively vigorous growth at 30 °C relative to the other isolates, maintaining a floccose to cottony texture and well-developed mycelium, whereas isolates such as EMH7, LES19, and LES21 showed markedly reduced colony diameter and more compact growth under elevated temperature conditions. No diffusible pigments or exudates were observed in any isolate under the tested conditions.

**Figure 4:**
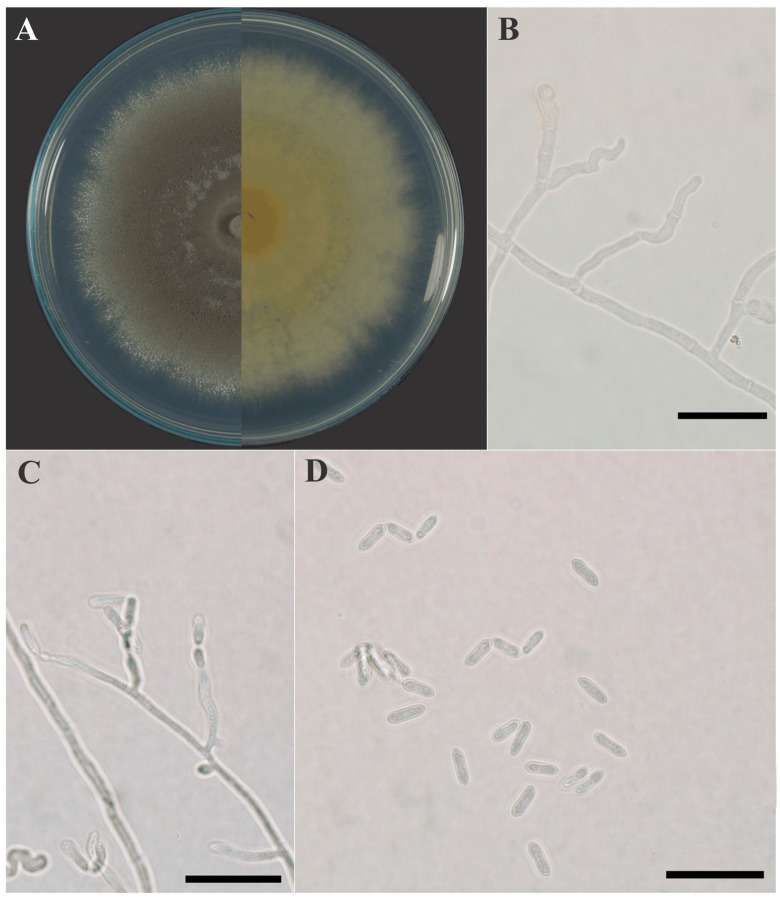
Micromorphological characteristics of *Metarhizium brunneum* isolate PM9. (**A**) Colony morphology on PDA showing obverse (**left**) and reverse (**right**) views after incubation at 25 °C. (**B**,**C**) Hyaline, septate hyphae with conidiogenous cells arising laterally. (**D**) Ellipsoidal to cylindrical conidia arranged singly or in short chains. Scale bars = 200 µm.

Microscopic observations, performed on isolate PM9 as a representative strain, revealed typical morphological features of *Metarhizium brunneum*, including hyaline, septate hyphae and conidiogenous cells producing ellipsoidal to cylindrical conidia (Figure 4). Conidia were observed singly or in short chains, with an average size of 7.37 ± 0.60 × 2.33 ± 0.19 µm.

#### 3.2.2. Physiological Characterization

Significant differences in mycelial growth were observed among isolates at both temperatures evaluated (*p* < 0.0001; Table 3). At 25 °C, isolates EMH7, LES19, LES21, and PM9 exhibited the highest radial growth values (29.34–30.26 mm), without significant differences among them (*p* < 0.05). EMH1 showed intermediate growth (24.98 mm), whereas PM6 presented the lowest value (19.67 mm). At 30 °C, PM6 displayed the greatest radial growth (22.16 mm), significantly higher than all other isolates (*p* < 0.05). EMH1 showed intermediate growth (17.99 mm), while LES19 exhibited the lowest value (4.78 mm).

Thermal sensitivity differed significantly among isolates (*p* < 0.0001). Most isolates showed growth inhibition at 30 °C relative to 25 °C, with LES19 presenting the highest inhibition (−83.74%), followed by LES21 (−73.22%) and PM9 (−61.88%). In contrast, PM6 exhibited positive growth stimulation (12.65%), indicating increased radial growth at 30 °C. Conidial production on PDA also differed significantly among isolates (*p* < 0.0001). EMH7 and LES21 produced the highest concentrations (1.05 × 10^8^ and 9.77 × 10^7^ conidia·mL^−1^, respectively), whereas LES19 showed the lowest production (5.17 × 10^7^ conidia·mL^−1^).

### 3.3. Conidial Production on Rice

**Figure 5:**
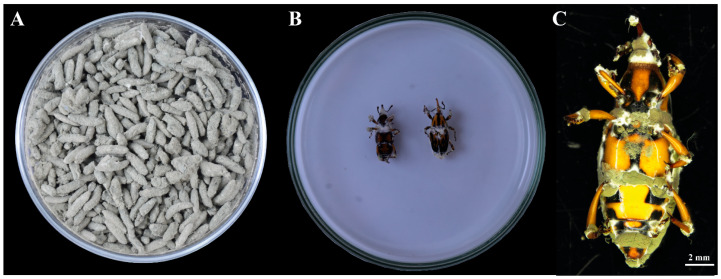
Growth and conidial production of *M. brunneum* isolate PM9. (**A**) Conidial production on rice substrate following solid-state fermentation at 25 ± 1 °C, illustrating abundant sporulation and substrate colonization. (**B**) Sporulation test on dead insects. (**C**) *M. hemipterus* adult with *M. brunneum* sporulate.

Significant differences in conidial production on rice were observed among isolates (*p* < 0.0001; Table 3). Isolate PM9 exhibited the highest conidial yield (2.63 × 10^9^ conidia·g^−1^), significantly exceeding all other isolates. EMH1 showed intermediate production (2.11 × 10^9^ conidia·g^−1^), followed by PM6 (1.76 × 10^9^ conidia·g^−1^). Lower yields were recorded for EMH7 and Les21 (1.65 × 10^9^ and 1.64 × 10^9^ conidia·g^−1^, respectively), whereas LES19 presented the lowest production (1.12 × 10^9^ conidia·g^−1^). In contrast to the pattern observed on PDA, conidial production on rice did not follow the same ranking among isolates. Notably, although EMH7 and LES21 showed high sporulation on PDA, their yields on rice were comparatively lower. Conversely, PM9 displayed moderate production on PDA but the highest conidial yield on rice substrate (Figure 5).

The results indicate that conidial production varied according to the culture substrate. While significant differences among isolates were observed on both PDA and rice, the relative performance of isolates was not consistent across substrates. This suggests substrate-dependent variation in sporulation capacity among *M. brunneum* isolates.

### 3.4. Dose–Response Curves in Banana Weevil Adults

**Figure 6:**
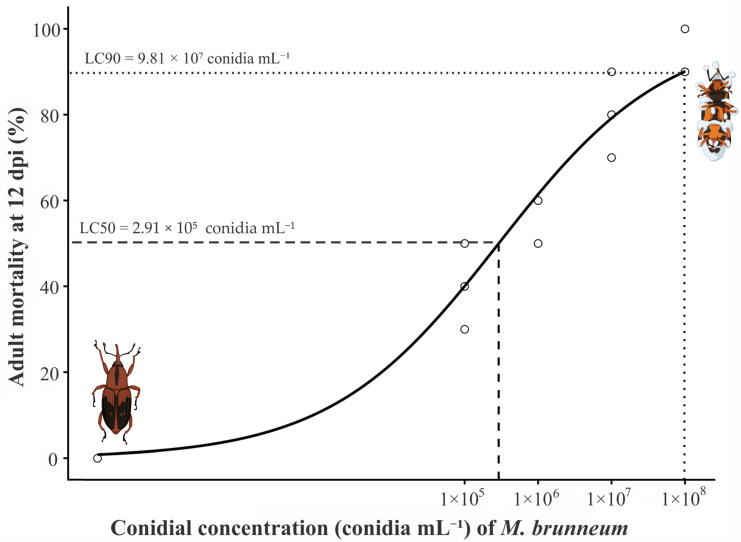
Dose–response curve of *M. brunneum* (isolate PM9) against adults of *M. hemipterus* at 12 days post-inoculation (dpi). Points represent observed mortality values, and the solid line corresponds to the fitted four-parameter log-logistic model. Vertical dashed lines indicate the estimated LC_50_ and LC_90_ values.

The dose–response bioassay revealed a concentration-dependent of isolated PM9 increase in adult mortality of *M. hemipterus* (*p* < 0.0001; Figure 6). After 12 days post-inoculation of isolated Pm9, mortality ranged from 42.5 ± 9.6% at 1 × 10^5^ conidia·mL^−1^ to 92.5 ± 5.0% at 1 × 10^8^ conidia·mL^−1^. Mycosis was confirmed by external fungal sporulation on cadavers, verifying that mortality resulted from fungal infection. Based on the mortality data, the median lethal concentration (LC_50_) was estimated at 2.91 × 10^5^ ± 7.90 × 10^4^ conidia·mL^−1^, whereas the LC_90_ was 9.81 × 10^7^ ± 5.82 × 10^7^ conidia·mL^−1^. The four-parameter log-logistic model was statistically significant (*p* = 0.00224), with an R^2^ of 0.933 and an RMSE of 7.02.

## 4. Discussion

The development of biopesticides based on entomopathogenic fungi has received increasing attention as a sustainable alternative to chemical insecticides [21,34]. Among these microorganisms, species of *Metarhizium* are widely recognized for their potential in the management of agricultural pests due to their broad host range and capacity to persist in soil and plant-associated environments [7,8,35]. Despite this potential, relatively few studies have investigated naturally occurring entomopathogenic fungi infecting banana weevils under field conditions.

The present study documents the natural infection of *Metamasius hemipterus* by *Metarhizium brunneum* in banana plantations of the Amazonas region, Peru, and demonstrates that isolate PM9 combines robust taxonomic identity, physiological competence, and high virulence under laboratory conditions. By integrating multilocus phylogenetic analysis, physiological characterization, and dose–response modeling, this work links species-level identification with functional traits relevant to biological control. The natural occurrence of this pathogen in field populations is particularly significant, as indigenous strains are often better adapted to local environmental conditions and host populations than introduced isolates [2,36].

Multilocus phylogenetic reconstruction based on concatenated TEF-1α, β-tubulin, RPB1, and RPB2 sequences placed all isolates within the *M. brunneum* clade, with strong bootstrap support (≥96%) at the relevant nodes. The isolates clustered together with reference strain ARSEF 4179 and the ex-type strain ARSEF 2107T, confirming their taxonomic identity. No internal phylogenetic structuring was observed among the isolates, indicating limited genetic divergence at the loci analyzed (Figure 2).

The use of a multilocus framework is particularly important within the genus Metarhizium, where morphological similarity and cryptic speciation can obscure species boundaries when relying on single-gene markers [37,38]. Accurate species-level identification is not merely taxonomic but functionally relevant, as members of the *M. anisopliae* species complex may differ in host range, ecological fitness, and environmental tolerance [39]. Therefore, the robust phylogenetic placement of the present isolates provides a reliable foundation for interpreting their physiological and pathogenic traits.

Studies on the diversity and global distribution of *Metarhizium* have shown that species within this genus can occur across wide geographic regions while remaining genetically closely related [2,4]. The limited genetic divergence observed among the isolates in the present study is consistent with this pattern and may reflect the broad ecological adaptability of *M. brunneum*, a species frequently associated with soil environments and diverse insect hosts. Previous surveys have also reported *M. brunneum* as one of the most frequently recovered species in soil environments, with its distribution influenced by environmental factors such as soil carbon content, C:N ratio, and microbial activity [3].

Marked physiological differences were observed among isolates, particularly in radial growth and thermal sensitivity (Table 3). While most isolates exhibited reduced growth at 30 °C relative to 25 °C, isolate PM6 showed enhanced growth under higher temperature conditions, indicating intraspecific variability in thermal response. Temperature is a major ecological driver of fungal development and host infection processes [5], and variation in thermal tolerance has been widely reported among *Metarhizium* isolates [3]. Such heterogeneity highlights the importance of considering environmental adaptability when selecting strains for biological control applications.

The observed differences in radial growth were accompanied by subtle but consistent changes in colony morphology across temperatures (Table 3, Figure 3). At 25 °C, colonies generally exhibited floccose to cottony textures with abundant aerial mycelium, whereas growth at 30 °C resulted in more compact colonies with reduced aerial development. Temperature-associated morphological plasticity has been reported in *Metarhizium* spp. and reflects adaptive responses influencing sporulation dynamics and tolerance to environmental stress [2,3,5].

Similar patterns of thermal growth inhibition have been reported in other studies of *Metarhizium brunneum*. For example, Mayo-Hernández et al. [22] observed that isolates grown at 30 °C exhibited reduced radial growth compared with 25 °C, with colony diameters ranging from 5.17 to 5.81 mm and an overall reduction of 64.7–69.1% in growth across isolates. In contrast, Torres-de la Cruz et al. [32] reported lower inhibition levels (0–27%) in native isolates of *M. anisopliae*, highlighting interspecific variability in thermal responses among entomopathogenic fungi. Previous studies have also indicated that the optimal temperature range for most entomopathogenic fungi lies between 20 and 28 °C [40]. Mycelial growth under different temperature conditions is therefore considered an important parameter when selecting promising isolates for biological control programs [41].

Conidial production varied significantly among isolates and differed according to the substrate used (Table 3). Notably, the ranking of isolates on PDA did not correspond to their performance on rice substrate. While some isolates exhibited high sporulation on artificial agar medium, their conidial yield on rice was comparatively lower. In contrast, isolate PM9 displayed moderate production on PDA but the highest yield on rice (Table 3, Figure 5). Substrate-dependent sporulation capacity has been documented in entomopathogenic fungi and may reflect differences in nutrient utilization efficiency and colonization dynamics [42]. Similar observations have been reported by Mayo-Hernández et al. [22], who found that Metarhizium isolates showing high sporulation on artificial media did not necessarily exhibit the highest conidial production on rice substrates used for solid-state fermentation.

From an applied standpoint, rice-based solid-state fermentation systems are widely employed for large-scale production of *Metarhizium* spp. due to their low cost and high conidial yield [43,44]. Consequently, the high conidial production observed for isolate PM9 under these conditions represents an advantageous trait for potential commercial development. Conidial yield is a key parameter in the selection of promising isolates for biological control programs, as it directly influences formulation efficiency and field applicability [22,45,46].

The pathogenicity assays confirmed the strong virulence of *Metarhizium brunneum* isolate PM9 against adults of *Metamasius hemipterus*. The LC_50_ estimated for PM9 (2.91 × 10^5^ conidia·mL^−1^; Figure 5) indicates a high level of infectivity under laboratory conditions. This value is substantially lower than LC_50_ estimates reported for other entomopathogenic fungi evaluated against *M. hemipterus*, where values ranging from 2.04 × 10^9^ to 7.94 × 10^11^ conidia·mL^−1^ have been reported for *Metarhizium anisopliae*, *Metarhizium* sp., and *Beauveria peruvienensis* [21]. Such differences may reflect intrinsic variation in virulence among fungal species and isolates, as well as methodological differences among bioassays.

High mortality levels caused by *Metarhizium* isolates have also been documented in other insect hosts. For example, Mayo-Hernández et al. [22] reported mortality rates of up to 96.4% and 89.7% in *Antiteuchus tripterus* eight days after inoculation with highly virulent isolates. Similarly, Resquín-Romero et al. [47] observed mortality ranging from 83.3% to 100% in nymphs and adults of *Euschistus heros* exposed to *M. brunneum*. Other studies have also demonstrated the pathogenic potential of *M. brunneum* against a wide range of insect pests, including *Bactrocera oleae* and *Agriotes* spp. [35,48]. Likewise, isolates of *M. anisopliae* have caused high mortality levels in hemipteran pests such as *Nezara viridula* and *Dichelops melacanthus* [49,50], highlighting the broad applicability of entomopathogenic fungi in insect pest management.

In banana agroecosystems, entomopathogenic fungi have also been evaluated against coleopteran pests associated with the crop. For instance, Negrete González et al. [51] reported that isolate Ma148 of *M. anisopliae* caused 76.9% mortality in adults of the banana weevil *Cosmopolites sordidus*, with an LC_50_ of 8.6 × 10^6^ conidia·mL^−1^ under laboratory conditions. Compared with these results, the lower LC_50_ obtained for PM9 suggests a comparatively higher virulence of this isolate against *M. hemipterus*.

The sigmoidal response pattern observed (Figure 6) is consistent with the infection dynamics of entomopathogenic fungi, in which increasing propagule density enhances host–pathogen contact probability and successful cuticular penetration [5]. Although LC_90_ values exhibited greater variability, mortality at the highest concentration exceeded 90%, and mycosis was confirmed by external sporulation, ensuring that mortality was attributable to fungal infection rather than handling effects. Similar infection dynamics have been reported in other studies involving *M. brunneum*. For example, Zottele et al. [10] reported that direct treatment of beetles with 5 × 10^6^ conidia per individual resulted in LT_50_ and LT_90_ values of 5.5 and 10.2 days, respectively, under laboratory conditions. In that study, fungal outgrowth was also consistently observed on cadavers after death, confirming that mortality resulted from fungal infection.

The selection of effective entomopathogenic fungi for biological control requires consideration of multiple attributes beyond virulence alone, including inoculum production, growth capacity, sporulation efficiency, and tolerance to environmental stress [8,44,52]. In this context, the integration of physiological and pathogenicity data in the present study identified isolate PM9 as a promising candidate despite not exhibiting the highest radial growth rate. The combination of high conidial production on rice substrate and strong virulence therefore enhances the practical relevance of this isolate for biological control applications.

In banana production systems, management of weevil pests relies primarily on cultural practices, pheromone-based trapping, and, in some cases, chemical insecticides. However, chemical control is often limited by environmental concerns, residue restrictions, and reduced efficacy due to the concealed feeding habits of larvae within pseudostem tissues. Consequently, biological control strategies have gained increasing attention as sustainable alternatives [20,51].

Entomopathogenic fungi such as *Beauveria bassiana* and *Metarhizium anisopliae* have been evaluated against banana weevils and other curculionid pests, with variable success depending on environmental conditions and formulation [36,51,53]. Within this context, the identification of a naturally occurring and locally adapted *M. brunneum* isolate offers potential advantages. Integration of such fungal agents with pheromone trapping systems or cultural sanitation practices could enhance suppression of adult populations while reducing reliance on chemical inputs. The compatibility of *Metarhizium* spp. with integrated pest management (IPM) programs has been demonstrated in several cropping systems [8,14,15], supporting the feasibility of incorporating isolate PM9 into biologically based management strategies.

Nevertheless, the present study was conducted under controlled laboratory conditions, which may not fully represent the environmental variability encountered in field settings. Factors such as temperature fluctuations, UV radiation, rainfall, and interactions with native microbial communities can strongly influence fungal persistence and efficacy [9,13,51]. Additionally, only adult stages were evaluated, and susceptibility may differ among larval or pupal stages of *M. hemipterus*. Future research should therefore assess field performance, environmental persistence, formulation stability, and compatibility with other IPM components, including cultural practices and semiochemical-based monitoring systems, to determine the operational potential of this isolate within sustainable banana production systems.

## 5. Conclusions

This study documents the natural infection of *Metamasius hemipterus* by *Metarhizium brunneum* in banana agroecosystems of the Amazonas region, Peru. Multilocus phylogenetic analysis confirmed the taxonomic identity of the isolates within the *M. brunneum* clade, providing a robust framework for interpreting their biological traits. Among the evaluated isolates, PM9 exhibited the most promising combination of characteristics, including high conidial production on rice substrate and strong pathogenicity against adult weevils. The relatively low LC_50_ value obtained under laboratory conditions indicates high infectivity and suggests strong host compatibility.

## Figures and Tables

**Table 1:** Collection sites of *M. hemipterus* individuals naturally infected with *M. brunneum* in the Amazonas region, Peru.

Isolate	Variety	Province	District	Latitude (°S)	Longitude (°W)	Life Stage
EMH1	Valery	Utcubamba	Bagua Grande	−5.904444	−78.329722	Pupa
EMH7	Isla	Utcubamba	Bagua Grande	−5.883333	−78.317222	Adult
LES19	Bellaco	Rodríguez de Mendoza	Huambo	−6.431324	−77.536046	Larva
LES21	Seda	Rodríguez de Mendoza	Omia	−6.422469	−77.335473	Adult
PM6	Bellaco	Bongara	Valera	−6.069061	−77.903197	Larva
PM9	Bellaco	Bongará	Pedro Ruiz	−5.915421	−77.956741	Larva

**Table 2:** Reference strains of *Metarhizium* species included in the multilocus phylogenetic analysis, with GenBank accession numbers for TEF-1α, β-TUB, RPB1, and RPB2 sequences.

Species	Host	Country	Isolate	TEF-1α	β-TUB	RPB1	RPB2
*M. mendoncearum*	*M. posticata*	Brazil	URM 8140	MZ394805	MZ394811	MW885972	MZ394799
*M. mendoncearum*	*A. alveata*	Brazil	URM 8143T	MZ394808	MZ394814	MW885975	MZ394802
*M. acridum*	Orthoptera	Niger	ARSEF 7486T	EU248845	EU248813	EU248897	EU248925
*M. acridum*	Orthoptera	Australia	ARSEF 324	EU248844	EU248812	EU248896	XM_066120576
*M. alvesii*	Soil	Brazil	CG1123T	KY007614	KY007611	KY007612	KY007613
*M. anisopliae*	Orthoptera	Eritrea	ARSEF 7487T	DQ463996	EU248822	DQ468355	DQ468370
*M. anisopliae*	Coleoptera	Australia	ARSEF 7450	EU248852	EU248823	EU248904	EU248932
*M. brunneum*	Coleoptera	USA	ARSEF 2107T	EU248855	EU248826	EU248907	EU248935
*M. brunneum*	Soil	Australia	ARSEF 4179	EU248854	EU248825	EU248906	EU248934
*M. brunneum*	*M. hemipterus*	*Peru*	*EMH1*	PZ371316	PZ353158	PZ353146	PZ353152
*M. brunneum*	*M. hemipterus*	Peru	EMH7	PZ371317	PZ353159	PZ353147	PZ353153
*M. brunneum*	*M. hemipterus*	Peru	LES19	PZ371320	PZ353162	PZ353150	PZ353156
*M. brunneum*	*M. hemipterus*	Peru	LES21	PZ371318	PZ353163	PZ353151	PZ353157
*M. brunneum*	*M. hemipterus*	Peru	PM6	PZ371321	PZ353160	PZ353148	PZ353154
*M. brunneum*	*M. hemipterus*	Peru	PM9	PZ371319	PZ353161	PZ353149	PZ353155
*M. frigidum*	Coleoptera	Australia	ARSEF 4124T	DQ464002	EU248828	DQ468361	DQ468376
*M. globosum*	Lepidoptera	India	ARSEF 2596T	EU248846	EU248814	EU248898	EU248926
*M. guizhouense*	Lepidoptera	China	CBS 258.90T	EU248862	EU248834	EU248914	EU248942
*M. guizhouense*	Lepidoptera	China	ARSEF 6238	EU248857	EU248830	EU248909	EU248937
*M. humberi*	Soil	Brazil	IP 46T	MH837574	MH837547	MH837556	MH837565
*M. humberi*	Soil	Brazil	IP 151	MH837579	MH837552	MH837561	MH837570
*M. indigoticum*	Lepidoptera	Japan	NBRC 100684T	KJ398784	KJ398544	OR115266	KJ398692
*M. lepidiotae*	Coleoptera	Australia	ARSEF 7488T	EU248865	EU248837	EU248917	EU248945
*M. lepidiotae*	Coleoptera	Australia	ARSEF 7412	EU248864	EU248836	EU248916	EU248944
*M. majus*	Coleoptera	Philippines	ARSEF 1946	EU248867	EU248839	EU248919	EU248947
*M. pinghaense*	Coleoptera	China	CBS 257.90T	EU248850	EU248820	EU248902	EU248930
*M. pinghaense*	Coleoptera	Solomon Islands	ARSEF 4342	EU248851	EU248821	EU248903	EU248931
*M. robertsii*	—	Australia	ARSEF 7501	EU248849	EU248818	EU248901	EU248929
*M. robertsii*	Orthoptera	Brazil	ARSEF 727	DQ463994	EU248816	DQ468353	DQ468368

Type strains are indicated by the suffix “T”. Accession numbers correspond to sequences retrieved from GenBank. The dataset includes representative species of the *M. anisopliae* species complex. Outgroup: *M. frigidum* (ARSEF 4124T).

**Table 3:** Physiological characteristics of *Metarhizium brunneum* isolates, including mycelial growth at 25 and 30 °C, thermal sensitivity, and conidial production on PDA and rice substrate.

Isolate	Mycelial Growth (mm)		Thermal Sensitivity (%)	Conidial Production on PDA (conidia·mL^−1^)	Conidial Production on Rice (conidia·g^−1^)
	25 °C	30 °C			
EMH1	24.98 ± 0.57 b	17.99 ± 0.94 b	−27.88 ± 4.98 b	6.43 × 10^7^ ± 3.53 × 10^6^ d	2.11 × 10^9^ ± 1.01 × 10^8^ b
EMH7	29.81 ± 1.16 a	12.53 ± 0.24 c	−57.95 ± 1.52 c	1.05 × 10^8^ ± 2.82 × 10^6^ a	1.65 × 10^9^ ± 1.59 × 10^8^ d
LES19	29.41 ± 0.62 a	4.78 ± 0.28 f	−83.74 ± 0.93 e	5.17 × 10^7^ ± 8.72 × 10^6^ e	1.12 × 10^9^ ± 1.07 × 10^8^ e
LES21	30.26 ± 1.04 a	8.09 ± 0.10 e	−73.22 ± 1.13 d	9.77 × 10^7^ ± 2.96 × 10^6^ a	1.64 × 10^9^ ± 1.39 × 10^8^ d
PM6	19.67 ± 0.25 c	22.16 ± 0.36 a	12.65 ± 0.91 a	8.04 × 10^7^ ± 1.51 × 10^6^ c	1.76 × 10^9^ ± 7.22 × 10^7^ c
PM9	29.34 ± 1.07 a	11.18 ± 0.13 d	−61.88 ± 1.28 c	8.99 × 10^7^ ± 5.65 × 10^6^ b	2.63 × 10^9^ ± 1.71 × 10^8^ a
c.v.	3.13	3.45	4.73	3.97	5.98
LSD	1.91	0.99	5.17	7.27 × 10^6^	1.03 × 10^8^
*p*	<0.0001	<0.0001	<0.0001	<0.0001	<0.0001

Values represent mean ± standard deviation (*n* = 4). Different letters within each column indicate significant differences according to Tukey’s test (*p* < 0.05). c.v.: coefficient of variation. LSD: least significant difference.

## Data Availability

The datasets generated in this study are available in the NCBI repository (https://www.ncbi.nlm.nih.gov/), with accession numbers provided in the article. The original contributions presented in this study are included in the article. Further inquiries can be directed to the corresponding authors.
